# Code-modulated visual evoked potentials using fast stimulus presentation and spatiotemporal beamformer decoding

**DOI:** 10.1038/s41598-017-15373-x

**Published:** 2017-11-08

**Authors:** Benjamin Wittevrongel, Elia Van Wolputte, Marc M. Van Hulle

**Affiliations:** 1 Department of Neurosciences, KU Leuven, Leuven, Belgium; 2 Department of Computer Science, KU Leuven, Leuven, Belgium

## Abstract

When encoding visual targets using various lagged versions of a pseudorandom binary sequence of luminance changes, the EEG signal recorded over the viewer’s occipital pole exhibits so-called code-modulated visual evoked potentials (cVEPs), the phase lags of which can be tied to these targets. The cVEP paradigm has enjoyed interest in the brain-computer interfacing (BCI) community for the reported high information transfer rates (ITR, in bits/min). In this study, we introduce a novel decoding algorithm based on spatiotemporal beamforming, and show that this algorithm is able to accurately identify the gazed target. Especially for a small number of repetitions of the coding sequence, our beamforming approach significantly outperforms an optimised support vector machine (SVM)-based classifier, which is considered state-of-the-art in cVEP-based BCI. In addition to the traditional 60 Hz stimulus presentation rate for the coding sequence, we also explore the 120 Hz rate, and show that the latter enables faster communication, with a maximal median ITR of 172.87 bits/min. Finally, we also report on a transition effect in the EEG signal following the onset of the stimulus sequence, and recommend to exclude the first 150 ms of the trials from decoding when relying on a single presentation of the stimulus sequence.

## Introduction

Among the gamut of brain-computer interfacing (BCI) paradigms^1^, the code-modulated visual evoked potential (cVEP) has been reported to the yield one of the highest information transfer rates (ITRs)^2^. The cVEP paradigm defines a binary sequence of high and low stimulus intensities with unequal duty cycles, called the ‘code’, and uses for each selectable target a unique lagged version of this code^2–4^. As coding sequence, the m-sequence^5^ is often chosen because of its favourable autocorrelation properties^2^, amongst other properties^6^. M-sequences have also been applied in other fields such as fMRI^7^ and sensor technology^8^. Albeit rarely adopted, as an alternative to m-sequences, a periodic pseudorandom binary code has been described for cVEP^9^.

As far as we are aware, only one group previously investigated the application of a faster stimulus presentation for cVEP (i.e., higher than the traditional 60 Hz)^10–13^. The encoding sequence was presented to the subject by LEDs at a carrier frequency of 40 Hz controlled by an Arduino micro-controller, which would compare to a 80 Hz screen refresh rate. The authors investigated the effect of stimulation colour^10,13^, classifier kernels^11^ and filter bands^12^, but could not achieve a higher decoding performance for the faster stimulus rate. In one of their studies^11^, they report on the decoding performance with an increasing number of m-sequence repetitions, but did not consider the implication on the performance in terms of ITR.

Although cVEP has achieved among the highest ITR, the paradigm is considerably less studied compared to other visual BCI paradigms such as the P300 event-related potential (ERP) and the steady-state visual evoked potential (SSVEP) (see the review of Gao and coworkers^14^ for reference). Traditionally, cVEPs are decoded from electroencephalography (EEG) using a template matching algorithm^2^, canonical correlation analysis (CCA)^13^ or a combination^15,16^. BCIs adopting these algorithms have been proven successful in online settings, including EEG-based spelling applications^17^ and robot control^18,19^, and have also been applied in an intracranial EEG (iEEG) setting^20^. In recent research, the support vector machine (SVM) has been shown to identify targets more accurately than the traditional decoding algorithms^10^, with a linear kernel achieving in the highest accuracy^11^.

Recently, a spatiotemporal extension of the beamforming algorithm has been introduced in EEG-BCI and shown to yield promising results with EEG signals that have consistent spatial and temporal characteristics, such as the N400-^21^ and P300^22^ ERPs. With SSVEP, the combination of the spatiotemporal beamformer with a time-domain analysis^23,24^ proved successful in both offline^25^ and online^26^ settings.

The goal of this study is to assess the performance of the spatiotemporal beamforming algorithm for target identification when using cVEP-based encoding, and to compare the performance for both traditional (60 Hz) and high-speed (120 Hz) stimulus presentations.

## Methods

### Subjects

Seventeen subjects with normal or corrected-to-normal vision participated in the experiment (14 female, 13 right handed, aged 22.35 ± 2.9, ranging from 18 to 30 years old). Prior to the experiment, the subjects read and, when they agreed, signed an informed consent form approved by the ethical committee of our university hospital UZ Leuven. All subjects received a monetary reward for their participation. This study was carried out in accordance with the relevant guidelines and regulations.

### Experimental design

**Figure 1:**
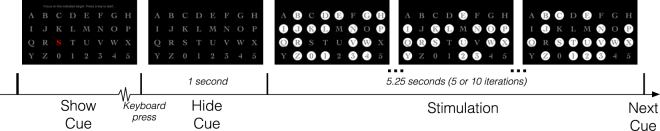
Time-course of one trial during the experiment.

The interface consisted of 32 circular white targets (4 cm diameter, 2 cm vertical and horizontal inter-target distance) that follow an m-sequence stimulation paradigm (see further) and that were overlaid with static (i.e., non-flickering) grey letters or numbers arranged in a matrix (Fig. 1). The interface was presented on a ViewPixx-EEG monitor (24 inch, native 120 Hz refresh rate, resolution of 1920 × 1080, VPixx Technologies, Canada). The subjects were seated approximately 60 cm from the monitor. At this distance, the circular targets spanned a visual angle of approximately 3.8°, with an inter-target angle of 1.9°. The experiment was implemented in Matlab, using the Psychophysics Toolbox extensions^27–29^.

The following m-sequence of length of 63 was used to encode the targets:

000100001011001010100100111100.000110111001100011101011111101101

where targets were lagged by integer multiples of two frames. We adopted the equivalent-neighbours strategy used in other studies^15,17^, but decided not to implement the additional outer border in order to reduce visual demand.

Figure 1 visualises the experimental interface during one trial. A trial started with the presentation of a cue (i.e., one target shown in red). Subjects were asked to redirect their gaze to the cued target and to press a button to start the stimulation. After that, all targets were hidden (with the characters still shown in grey) for one second, followed by the stimulation phase during which all targets adopted their unique lagged m-sequence and repeated this sequence either 5 or 10 times (depending on the session, see further). To avoid visual fatigue and boredom, subjects were allowed to take breaks between trials.

Unlike traditional 60 Hz monitors, the monitor used in our experiment had a refresh rate of 120 Hz, which allowed us to experiment with high-speed presentations of the coding sequence. The full experiment consisted of two sessions. In one session, *S*_120_, the stimulus presentation followed the screen refresh rate. In the other session, *S*_60_, we simulated the stimulation as it would be presented on a 60 Hz screen by presenting each entry of the m-sequence for two frames before moving to the next entry. In each trial of *S*_120_ and *S*_60_, the m-sequence was repeated 10 and 5 times, respectively. In both sessions, the stimulation duration per trial was 5.25 seconds, and all targets were cued 5 times in pseudorandom order, leading to a total of 160 (=32 × 5)trials per session.

The two sessions were counterbalanced across subjects: 9 of the 17 subjects started with *S*_60_, while the other 8 performed *S*_120_ first. Table 1 summarises the details of the two sessions.

**Table 1:** Stimulation and analysis details of both sessions.

Session	Stimulation			Analysis	
	stimulus presentation rate	m-sequence repetitions per trial	trial duration	downsampling	samples per segment
*S*_60_	60 Hz	5	5.25 s	100 Hz	105
*S*_120_	120 Hz	10	5.25 s	200 Hz	105

For the faster stimulation rate, the downsampling rate is doubled, leading to an equal number of samples in the segments of both sessions. Note that a segment corresponds to the EEG response elicited by one full presentation of the m-sequence.

### Recording

**Figure 2:**
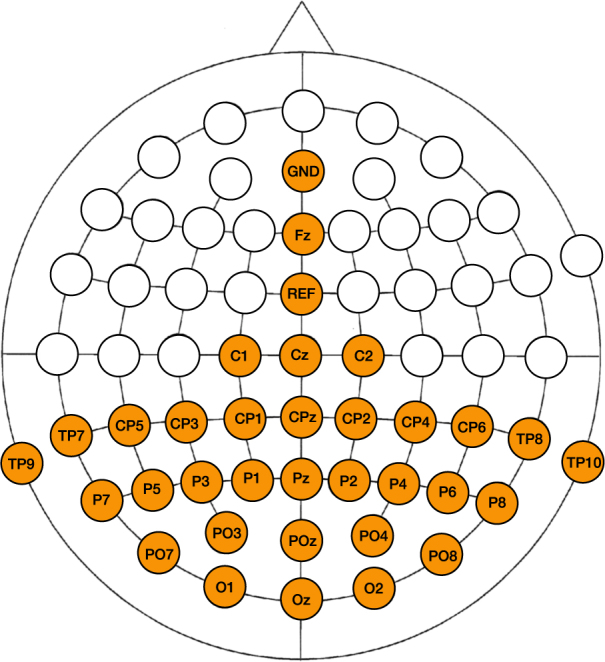
Locations of the 32 electrodes used during the experiment.

EEG was recorded continuously using a SynampsRT device (Compumedics Neuroscan, Australia) operating at a sampling rate of 2000 Hz with 32 active Ag/AgCl electrodes covering the parietal and occipital poles, where consistent activations in response to cVEP stimulation are expected^10,15^. The ground (GND) and reference (REF) electrodes were located at AFz and FCz, respectively (Fig. 2). Conductive gel was applied at each electrode site and impedances were kept below 2 kΩ.

### Preprocessing

The raw signal was re-referenced offline to the average of both mastoids signals (TP9 and TP10) and filtered between 4 and 31 Hz using a 4th order Butterworth filter, in order to attenuate the presence of artefacts such as slow drifts due to electrode gel expiration and sweat, low frequency oscillations due to electrode movements, high-frequency extraphysiologic noise, and powerline interference. The EEG was then cut into 5.25-second epochs starting from the onset of the stimulation, and labeled with the corresponding cued target. Finally, the epochs of *S*_60_ and *S*_120_ were downsampled to 100 Hz and 200 Hz, respectively, and stored for further analysis. The difference in downsampling rate was included to obtain a fair comparison between the classification results (each repetition of the m-sequence at both the traditional and faster stimulus rate has an equal number of samples, see Table 1). For each subject and session, 160 labeled epochs were extracted and saved.

### Classification

Target identification was achieved using a classifier based on the linearly-constrained minimum-variance (LCMV) spatiotemporal beamformer^21^. This recent extension of the original spatial beamformer estimates the contribution of an a-priori specified activation pattern (i.e., a template, a signal of interest) to the current input. It has been shown that LCMV beamforming is a special case of Minimum Variance Distortionless Response (MVDR) beamforming^30^, introduced to improve the robustness of the latter^31^. The EEG responses to the stimuli of interest are not only confluenced by ongoing brain activity but can also be modulated by the subject’s attention level, motivation and fatigue. The LCMV beamformer in an EEG context has shown to be effective as spatial filter for ERP detection^32^ and source localisation for studying source connectivity^33–35^, and its spatiotemporal extension has shown effective for ERP analysis^21^ and as target identification algorithm in BCI settings^22,25,26^.

**Figure 3:**
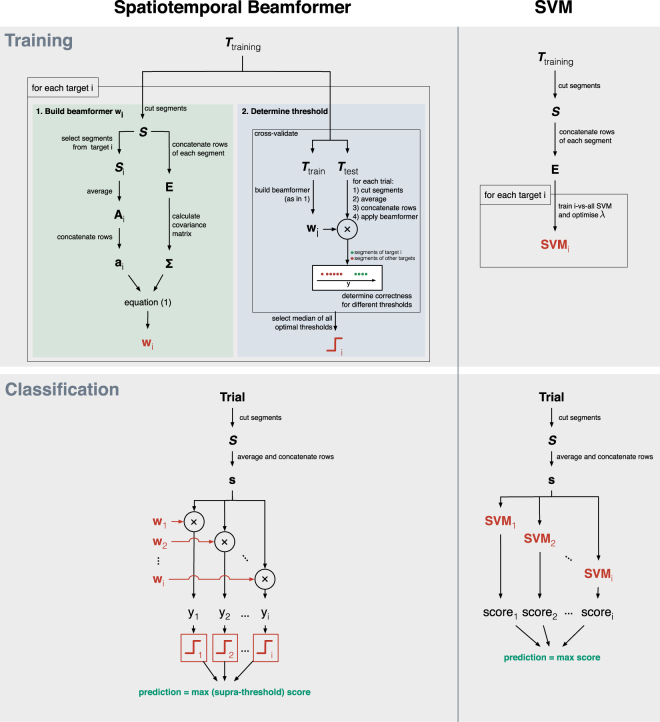
Visual representation of the training and classification procedure for the beamformer- and SVM-based classifier.

Since each target elicits a different brain response (cf. unique lags of the m-sequence), each target evokes an unique EEG activation pattern, and training the classifier thus involves the estimation of 32 activation patterns, each used to construct a beamformer tailored to a specific target. The training and classification procedures for both the beamformer- and SVM-based classifiers are depicted in Fig. 3.

#### Beamforming

The activation patterns and the beamformers (one for each target) were calculated from the training data ${T}_{training}\in {{\mathbb{R}}}^{m\times t\times l}$, where *m* is the number of channels, *t* is the number of samples and *l* is the number of epochs, as follows. For each epoch in ${T}_{training}$, a maximal number of *c*-second consecutive non-overlapping segments were extracted, where *c* represents the time needed to display one complete m-sequence. Let ${\boldsymbol{S}}\in {{\mathbb{R}}}^{m\times n\times r}$ be all *r* segments extracted and ${{\boldsymbol{S}}}_{i}\in {{\mathbb{R}}}^{m\times n\times k}$ be the segments from ***S*** in response to the cued target $i\in \mathrm{[1..32]}$, with *n* the number of samples per segment and *k* the total number of segments extracted for target *i*. Note that, while the m-sequences of *S*_120_ only span half the time ($c=0.525$ s) compared to *S*_60_ ($c=1.05$ s), their sampling rate is doubled so that the segments obtained from epochs of both *S*_60_ and *S*_120_ have the same number of samples.

The spatiotemporal activation pattern ${{\bf{A}}}_{i}\in {{\mathbb{R}}}^{m\times n}$ for target *i* was then obtained as the average of all *k* segments from ***S***_***i***_. The spatiotemporal beamformer ${{\bf{w}}}_{i}\in {{\mathbb{R}}}^{(mn)\times 1}$ for target *i* was calculated as an LCMV beamformer as follows: let ${\bf{E}}\in {{\mathbb{R}}}^{r\times (mn)}$ be the matrix where each row is obtained by concatenating the rows of a corresponding sequence ${\boldsymbol{S}}[\ast ,\ast ,r]$, $\Sigma \in {{\mathbb{R}}}^{(mn)\times (mn)}$ the covariance matrix of **E**, and ${{\bf{a}}}_{{\bf{i}}}^{{\rm{T}}}\in {{\mathbb{R}}}^{1\times (mn)}$ a vector containing the concatenated rows of **A**_***i***_. The LCMV beamformer under constraint ${{\bf{a}}}_{i}^{{\bf{T}}}{{\bf{w}}}_{i}=1$ can be calculated using the method of Langrage multipliers^36^:

1 $${{\bf{w}}}_{{\bf{i}}}=\frac{{{\rm{\Sigma }}}^{-1}{{\bf{a}}}_{{i}}}{{{\bf{a}}}_{{i}}^{{\rm{T}}}{{\rm{\Sigma }}}^{-1}{{\bf{a}}}_{{i}}}$$

and applied to the data as a simple weighted sum: ${y}_{i}={\bf{s}}{{\bf{w}}}_{i}$, where ${\bf{s}}\in {{\mathbb{R}}}^{1\times (mn)}$ indicates the concatenated rows of an input segment ${{\bf{S}}}_{in}\in {{\mathbb{R}}}^{m\times n}$.

In our study, the covariance matrix Σ was estimated using Matlab’s (2015a) *cov* function and was inverted using the *pinv* function, which calculates the Moore-Penrose pseudoinverse, to account for possible singularity of Σ.

In some studies, a single activation pattern **A**_1_ was calculated based on the EEG response to target 1, and the activation pattern **A**_*i*_ of target *i* was constructed as a circular-shifted version of **A**_1_ (following the phase difference between the m-sequences of targets 1 and *i*)^2,15^. However, given the availability of training data for each target, we opted to calculate the activation patterns for each target independently. In this way, discontinuities introduced by the circular shift were avoided and minor variations between templates were taken into account, leading to more accurate beamformers.

#### Classifier

In addition to building a beamformer for each target, a threshold was determined for each target in order to classify segments (in a one-vs-all fashion) into target-(positive class) and non-target (negative class). The threshold for each target was optimised via a Receiver Operating Characteristic (ROC) analysis^37,38^, using an additional 4-fold cross-validation on the training data (3 folds were used to train the beamformer, the remaining fold to test its performance). The ROC curve plots binary classification performance as a function of threshold value. Since the maximum classification performance could be reached for multiple thresholds (equal ROC points or points on the maximal iso-performance line), we selected the median of these.

Classification of a new epoch involved the extraction of the segments ${{\boldsymbol{S}}}_{test}$, using an identical procedure as for the training epochs. The segments were averaged and concatenated and then independently filtered by each beamformer to obtain a score *y*_*i*_ for each target *i*. Among the scores that exceeded the corresponding threshold, the one with the highest score was taken as winner. In case of none of the scores exceeded their threshold, the winner was determined by the highest (sub-threshold) score.

We compared our classifier based on spatiotemporal beamforming (stBF) with a SVM-based classifier. Similar to before, segments are extracted from the training epochs and concatenated to form feature vectors (cfr. ***E***). Then, for each target, a one-vs-all linear SVM^39^ was trained, whose regularisation parameter *λ* was optimised using a line-search strategy and 4-fold cross validation^40^. All SVMs were trained using the modified finite Newton method^41^. This procedure was successfully applied before to detect the P300 event-related potential (ERP) in patients with incomplete locked-in syndrome (LIS)^42^, to detect error-related potentials (ErrPs) in healthy subjects^43^, and served as a comparison for the spatiotemporal beamformer for P300 detection^22^. SVMs have been shown to outperform the traditional CCA classifier for cVEP detection^10,11^, and we opted for an optimised version of the SVMs in order to maximise accuracy. Prediction of a given (concatenated) input segment (cfr. ***s***) was given by the SVM returning the highest (i.e., most positive) score.

### Channel selection

For each subject, the channels included in the analysis were obtained using a greedy approach, in which we iteratively added the channel that improved the accuracy the most until it did no longer improve or until 100% accuracy was reached. As optimisation criterion, we used the classification accuracy obtained with the beamformer-based classifier when averaging two repetitions of the m-sequence (i.e., signal length of 2.10 and 1.05 sec for *S*_60_ and *S*_120_, respectively).

### Transition effect

It has been shown that the brain exhibits a latency of 100 to 150 ms in response to SSVEP stimulation^44,45^. During this time, the SSVEP is not stable, and in SSVEP-BCI research, the initial 100 to 150 ms of the epochs (time-locked to the onset of the flickering stimulation) is often excluded from analysis as it leads to increased accuracies^25,46^. Similar to SSVEP, cVEP is a visual paradigm adopting flickering stimulation (albeit not periodic), and we tested whether performance could be improved by excluding the initial 150 ms of each epoch. Note that, when excluding the first 150 ms of each epoch, an additional 150 ms is required at the end of the epoch to obtain the same number of complete m-sequences. For example, when excluding the initial signal, the first full m-sequence requires 0.150 + 1.05 = 1.2 seconds, compared to just 1.05 second without the exclusion.

In this study, we ran the analysis both with and without the exclusion of the initial 150 ms of each epoch.

### Performance evaluation

The performance of the classifiers was estimated offline using a stratified 5-fold cross-validation strategy. Since each target was cued 5 times, each fold contained one 5.25-second epoch for each target. We obtained the target identification accuracy for different signal lengths, corresponding to multiples of the time needed to present one repetition of the m-sequence.

As the two stimulus presentation rates as well as the possible exclusion of the initial signal lead to differences in stimulation time, one should be careful in interpreting the accuracies obtained by the different conditions. The ITR, however, takes into account the stimulation length and therefore provides a fair comparison between the conditions. Hence, next to target identification accuracy, we also measure ITR (in bits/min) as follows^47,48^:

2 $$ITR=\frac{{\mathrm{log}}_{2}N+p\mathrm{.}{\mathrm{log}}_{2}p+\mathrm{(1}-p\mathrm{).}{\mathrm{log}}_{2}(\frac{1-p}{N-1})}{t/60},$$

where *N* is the number of selectable targets, *p* is the accuracy of target identification, and *t* is the time needed to make a selection (in seconds). In our study, *N* was equal to 32 and *t* was set to the stimulation length plus an additional 500 ms to account for the time the subject would need to switch their gaze to the next target. In the literature, studies investigating BCI spelling interfaces often adopt a gaze-switching interval in the range from 300 to 1000 ms^17,46,49–51^, and a 500 ms interval has been shown feasible in an online setting^52^, albeit with the SSVEP paradigm.

In addition to accuracy and ITR, we also measured the time needed to train the spatiotemporal beamformer- and SVM-based classifiers on all data for each subject. Timings were collected on a quad-core 2.3 GHz Intel i7 machine.

### Statistics

Since the distributions do not consistently follow a gaussian distribution, we adopted the non-parametric (two-tailed) Wilcoxon signed rank test. We used this test to compare the accuracy of both classifiers and to compare the influence of excluding the first 150 ms of each epoch. The significance threshold was set to 0.05.

### Data Availability

The (anonymised and pre-processed) data that support the findings of this study, as well as the implementation of the classifiers and the analysis, are made available at https://kuleuven.box.com/v/CVEP.

## Results

**Figure 4:**
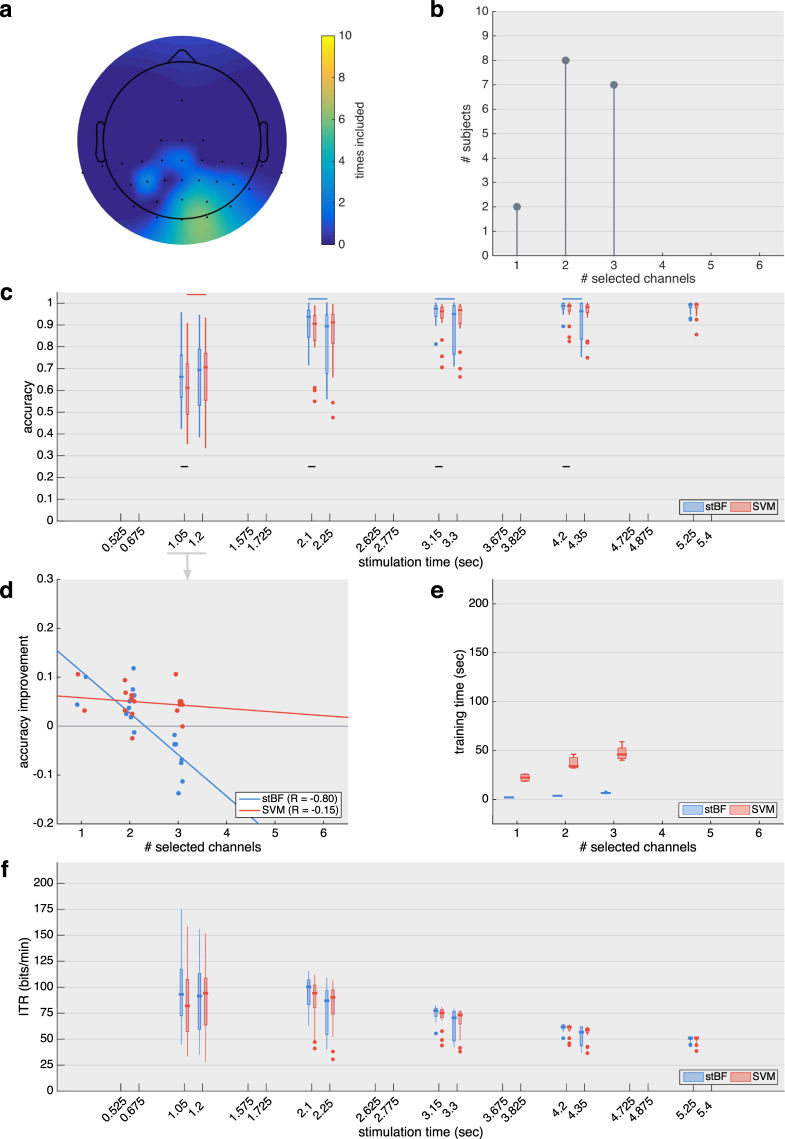
Results for the session adopting the traditional 60 Hz stimulus rate. (**a**) Scalp plot indicating how many times each channel was selected by the greedy channel-selection algorithm across subjects. Note that most of the frontal and temporal area was not recorded during the experiment. (**b**) Summary of the total number of channels selected by the greedy channel-selection algorithm before convergence. (**c**) Target identification accuracy for both classifiers with an increasing number of repetitions of the stimulation sequence (1 m-sequence = 1.05 sec), with and without the initial 150 ms of each epoch. Black horizontal lines indicate significant differences between the classifiers. Blue and red horizontal lines indicate significant differences when excluding the first 150 ms. (**d**) Regression analysis of the increase in target identification accuracy based on one repetition of the m-sequence when excluding the first 150 ms. (**e**) Time needed to train the classifiers on all data of each subject. (**f**) Virtual ITR achieved when factoring in 0.5 seconds for gaze shifting.

**Figure 5:**
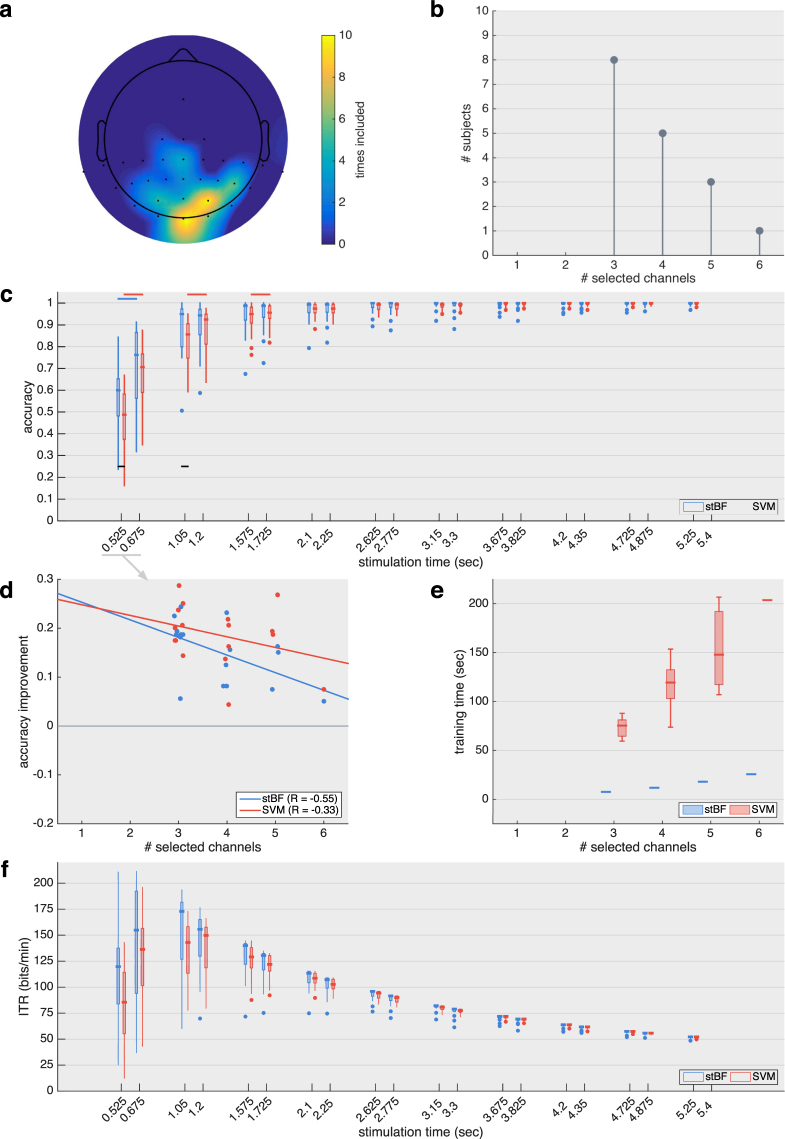
Results for the session adopting the 120 Hz stimulus rate. (**a**) Scalp plot indicating how many times each channel was selected by the greedy channel-selection algorithm across subjects. Note that most of the frontal and temporal area was not recorded during the experiment. (**b**) Summary of the total number of channels selected by the greedy channel-selection algorithm before convergence. (**c**) Target identification accuracy for both classifiers with an increasing number of repetitions of the stimulation m-sequence (1 sequence = 0.525 sec), with and without the exclusion of the initial 150 ms of each epoch. Black horizontal lines indicate significant differences between the classifiers. Blue and red horizontal lines indicate significant differences when excluding the first 150 ms. (**d**) Regression analysis of the increase in target identification accuracy based on one repetition of the m-sequence when excluding the first 150 ms. (**e**) Time needed to train the classifiers on all data of each subject. (**f**) Virtual ITR achieved when factoring in 0.5 seconds for gaze shifting.

All results of *S*_60_ and *S*_120_ are summarised in Figs 4 and 5, respectively.

For both sessions, the optimal channel set was obtained using a greedy approach. For *S*_60_, all subjects reached convergence with three or less channels (Fig. 4b), while between 3 and 6 channels were selected for *S*_120_ (Fig. 5b) before convergence was reached. The occipital channels Oz and O1 were selected most often (Figs 4a and 5a), and several parietal channels were selected by a smaller number of subjects, indicating considerable inter-subject variability.

Using the individually optimised channel sets, the target identification accuracy for both the spatiotemporal beamformer- and the SVM-based classifier are shown in Fig. 4c for *S*_60_ and Fig. 5c for *S*_120_, both with and without the exclusion of the initial 150 ms of the stimulation. As expected, longer stimulation times (i.e., more repetitions of the m-sequence) increases performance. For the same stimulation lengths, the faster stimulus presentation (*S*_120_) is able to present twice the amount of m-sequences compared to *S*_60_, which results in a higher accuracy for equal-length stimulation. Only the faster stimulus presentation in combination with the exclusion of the initial signal is able to surpass the accuracy threshold of 70% with a single repetition of the m-sequence. All other conditions require at least two repetitions to reach this threshold, which is deemed minimal for establishing reliable communication^42,53–55^.

Using the full signal, the accuracies of both classifiers differ significantly when averaging up to four (*S*_60_, Fig. 4c,) (*p* = 0.033; *p* = 0.012; *p* = 0.0017 and *p* = 0.030) and two (*S*_120_, Fig. 5c) (*p* = 0.016 and *p* = 0.020) repetitions of the m-sequence, respectively. With the exclusion of the initial 150 ms, the two classifiers are not significantly different. However, within stBF, the accuracies with and without the exclusion of the initial signal significantly differ for 2 to 4 repetitions (*S*_60_) (*p* < 0.001, *p* = 0.003 and *p* = 0.004) and 1 repetition (*S*_120_) ($p < 0.001$) of the m-sequence, respectively. Similarly, within the SVM-based classifier, the accuracies with and without the exclusion of the initial signal significantly differ for one repetition (*S*_60_) (*p* < 0.001) and 1 to 3 repetitions (*S*_120_) (*p* < 0.001, *p* < 0.001 and *p* = 0.008) of the m-sequence, respectively.

A detailed inspection of the accuracy increase with one repetition of the m-sequence (Fig. 4d for *S*_60_ and Fig. 5d for *S*_120_) shows a negative relation between the increase in accuracy and the number of selected channels. This effect is most prominent for stBF at the traditional 60 Hz stimulus rate (Fig. 4d). All subjects requiring three channels have a reduction in accuracy by removing the first 150 ms of the epochs, while the other subjects have an increased accuracy. The SVM is less influenced by the number of channels, and removing the initial 150 ms signal only decreases its accuracy for two subjects. While this negative trend can also be detected for the faster stimulation rate, all subjects have an increased accuracy compared to when the initial 150 ms signal is included in the analysis.

For both sessions, the time needed to train stBF on all data of each subject is significantly lower compared to SVM (Fig. 4e for *S*_60_ and Fig. 5e for *S*_120_), and for both classifiers, the training time increases when more channels are included in the analysis.

For both the traditional and faster stimulus presentation rates, the median ITR reaches its maximal value of 100.46 and 172.87 bits/min, respectively, using the beamformer-based classifier, two repetitions of the m-sequence and the full signal (stimulation time = 2.1 and 1.05 seconds, respectively).

## Discussion

In this study, we assessed the feasibility of spatiotemporal beamforming for resolving m-sequence encoded targets in a cVEP setting, and investigated the influence of stimulus presentation rate on target identification accuracy and ITR.

We showed that the proposed classifier is able to accurately discriminate targets, and that it is able to compete with a classifier based on optimised linear SVMs. We additionally show that a faster stimulus presentation rate is beneficial for the communication speed, as more iterations of the m-sequence can be presented in an equal amount of time. Both stimulation rates have similar performance in terms of number of m-sequence repetitions, and at least two repetitions are necessary to obtain a performance over 70%, which is deemed minimal for establishing reliable communication^42,53–55^. With two repetitions of the m-sequence, the median ITR is maximal and reaches 100.46 bits/min for the traditional 60 Hz and 172.87 bits/min for the faster 120 Hz stimulus presentation, respectively. As far as we are aware, no other cVEP study has reported a higher ITR. As commercial monitors with high frame rates are becoming increasingly more accessible at affordable prices, they are recommended for cVEP-BCI applications.

Compared to the SVM, the spatiotemporal beamformer can be trained considerably faster, as there are no parameters to be optimised. This could be important to achieve fast, online retraining of the beamformer-based classifier without causing the interface to be temporarily unavailable or to interfere with stimulation. The shorter training time would also allow for other optimisation algorithms to be executed (eg., channel selection, downsampling rate, filtering range, etc.) that would otherwise not be able to complete within a reasonable time.

We present evidence that the cVEP response exhibits a transition effect following the onset of a stimulation sequence. Previously, a response latency of 100 to 150 ms has been described for SSVEP^45^, and in recent SSVEP-BCI research, the initial signal was excluded from the analysis to improve target identification^25,46^. In this study, excluding the initial 150 ms of each epoch improves classification accuracy of both classifiers when using merely one repetition of the m-sequence. The performance increase is negatively correlated with the number of selected channels and mostly affects the spatiotemporal beamformer, even causing a performance decrease when adopting three channels at the traditional 60 Hz stimulus presentation rate. For the 120 Hz case, all accuracies increase despite larger channels sets. The discrepancy between these results could be due to the fact that, when excluding the initial signal from each epoch, the last m-sequence of each epoch is not complete, and the number of complete training segments is reduced by 20% for *S*_60_ compared to only 10% for *S*_120_. In order to maintain the same number of training segments, one could extend the stimulation of the training session by 150 ms. Additionally, the negative correlation between increase in accuracy and number of selected channels can be explained by the fact that the dimensions of the spatiotemporal beamformer increase linearly with the number of channels, thereby requiring more data to accurately estimate the covariance matrix^56,57^ (cf., the curse of dimensionality).

## Conclusion

In this study, we have shown that a classifier based on spatiotemporal beamforming is able to accurately discriminate targets encoded by an m-sequence, and could be employed in the context of cVEP BCI. We compared the traditional 60 Hz and the faster 120 Hz stimulus presentation rates, and found that the latter yields more accurate results for equal stimulation lengths, as the encoding sequence can be presented twice as many times as with the 60 Hz case. The maximal median ITR for both stimulus presentation rates and for two iterations of the m-sequence was 100.46 bits/min for the 60 Hz (stimulation time = 2.1 seconds) and 172.87 bits/min for the 120 Hz case rate (stimulation time = 1.05 seconds). We additionally described a transition effect following the onset of the stimulation, similar to SSVEP, and showed that removing the initial 150 ms of the epochs significantly improves classification accuracy when relying on only one repetition of the encoding sequence.

## References

[1] Nicolas-Alonso LF, Gomez-Gil J (2012). Brain computer interfaces, a review. Sensors.

[2] Bin G, Gao X, Wang Y, Hong B, Gao S (2009). Vep-based brain-computer interfaces: time, frequency, and code modulations [research frontier. IEEE Computational Intelligence Magazine.

[3] Sutter EE (1992). The brain response interface: communication through visually-induced electrical brain responses. Journal of Microcomputer Applications.

[4] Hanagata, J. & Momose, K. A method for detecting gazed target using visual evoked potentials elicited by pseudorandom stimuli. In *Proc. 5th Asia Pacific Conf. Medical and Biological Engineering and 11th Int. Conf. Biomedical Engineering (ICBME)* (2002).

[5] Zierler N (1959). Linear recurring sequences. Journal of the Society for Industrial and Applied Mathematics.

[6] Golomb, S. W. *et al*. *Shift register sequences* (Aegean Park Press, 1982).

[7] Buračas GT, Boynton GM (2002). Efficient design of event-related fmri experiments using m-sequences. NeuroImage.

[8] Sachs, J., Herrmann, R., Kmec, M., Helbig, M. & Schilling, K. Recent advances and applications of m-sequence based ultra-wideband sensors. 2007 IEEE International Conference on Ultra-Wideband, 10.1109/icuwb.2007.4380914(2007).

[9] Nakanishi, M. & Mitsukura, Y. Periodicity detection for bci based on periodic code modulation visual evoked potentials. In *Acoustics, Speech and Signal Processing (ICASSP)*, *2012 IEEE International Conference on*, 665–668 (IEEE, 2012).

[10] Aminaka, D., Makino, S. & Rutkowski, T. M. Classification accuracy improvement of chromatic and high–frequency code–modulated visual evoked potential–based bci. In *International Conference on Brain Informatics and Health*, 232–241 (Springer, 2015).

[11] Aminaka, D., Makino, S. & Rutkowski, T. M. Svm classification study of code-modulated visual evoked potentials. In *Signal and Information Processing Association Annual Summit and Conference (APSIPA)*, *2015 Asia-Pacific*, 1065–1070 (IEEE, 2015).

[12] Aminaka, D., Makino, S. & Rutkowski, T. M. Eeg filtering optimization for code–modulated chromatic visual evoked potential–based brain–computer interface. In Symbiotic Interaction, 1–6 (Springer, 2015).

[13] Aminaka, D., Makino, S. & Rutkowski, T. M. Chromatic and high-frequency cvep-based bci paradigm. In *Engineering in Medicine and Biology Society (EMBC)*, *2015 37th Annual International Conference of the IEEE*, 1906–1909(IEEE, 2015). 10.1109/EMBC.2015.7318755 26736655

[14] Gao S, Wang Y, Gao X, Hong B (2014). Visual and auditory brain computer interfaces. IEEE Transactions on Biomedical Engineering.

[15] Bin G (2011). A high-speed bci based on code modulation vep. Journal of Neural Engineering.

[16] Wei Q, Feng S, Lu Z (2016). Stimulus specificity of brain-computer interfaces based on code modulation visual evoked potentials. PloS one.

[17] Spüler M, Rosenstiel W, Bogdan M (2012). Online adaptation of a c-vep brain-computer interface(bci) based on error-related potentials and unsupervised learning. PLoS ONE.

[18] Kapeller, C. *et al*. A bci using vep for continuous control of a mobile robot. *2013 35th Annual International Conference of the IEEE Engineering in Medicine and Biology Society (EMBC)*, 10.1109/embc.2013.6610734 (2013) 10.1109/EMBC.2013.6610734 24110921

[19] Riechmann H, Finke A, Ritter H (2016). Using a cvep-based brain-computer interface to control a virtual agent. IEEE Transactions on Neural Systems and Rehabilitation Engineering.

[20] Kapeller, C. *et al*. An electrocorticographic bci using code-based vep for control in video applications: a single-subject study. *Frontiers in Systems Neuroscience* **8**, 10.3389/fnsys.2014.00139 (2014). 10.3389/fnsys.2014.00139 PMC4124519 25147509

[21] van Vliet M (2016). Single-trial erp component analysis using a spatiotemporal lcmv beamformer. IEEE Transactions on Biomedical Engineering.

[22] Wittevrongel B, Van Hulle MM (2016). Faster p300 classifier training using spatiotemporal beamforming. International Journal of Neural Systems.

[23] Luo A, Sullivan TJ (2010). A user-friendly ssvep-based brain–computer interface using a time-domain classifier. Journal of neural engineering.

[24] Manyakov, N. V., Chumerin, N., Combaz, A., Robben, A. & Van Hulle, M. M. Decoding ssvep responses using time domain classification. In *IJCCI (ICFC-ICNC)*, 376–380 (2010).

[25] Wittevrongel B, Van Hulle MM (2016). Frequency- and phase encoded ssvep using spatiotemporal beamforming. PLOS ONE.

[26] Wittevrongel, B. & Van Hulle, M. M. Hierarchical online ssvep spelling achieved with spatiotemporal beamforming. *2016**IEEE Statistical Signal Processing Workshop (SSP)*10.1109/ssp.2016.7551800 (2016).

[27] Brainard DH, Vision S (1997). The psychophysics toolbox. Spatial vision.

[28] Pelli DG (1997). The videotoolbox software for visual psychophysics: Transforming numbers into movies. Spatial vision.

[29] Kleiner M (2007). What’s new in psychtoolbox-3. Perception.

[30] Souden M, Benesty J, Affes S (2010). A study of the lcmv and mvdr noise reduction filters. IEEE Transactions on Signal Processing.

[31] Mu, P., Li, D., Yin, Q. & Guo, W. Robust mvdr beamforming based on covariance matrix reconstruction. *Science China Information Sciences* 1–12 (2013).

[32] Treder MS, Porbadnigk AK, Avarvand FS, Müller K-R, Blankertz B (2016). The lda beamformer: Optimal estimation of erp source time series using linear discriminant analysis. NeuroImage.

[33] Van Hoey G (1999). Beamforming techniques applied in eeg source analysis. Proc. ProRISC99.

[34] Belardinelli, P., Ortiz, E. & Braun, C. Source activity correlation effects on lcmv beamformers in a realistic measurement environment. *Computational and mathematical methods in medicine* **2012** (2012). 10.1155/2012/190513 PMC3351244 22611439

[35] Hong JH, Ahn M, Kim K, Jun SC (2013). Localization of coherent sources by simultaneous meg and eeg beamformer. Medical & biological engineering & computing.

[36] Van Veen BD, Van Drongelen W, Yuchtman M, Suzuki A (1997). Localization of brain electrical activity via linearly constrained minimum variance spatial filtering. IEEE Transactions on biomedical engineering.

[37] Bewick V, Cheek L, Ball J (2004). Statistics review 13: receiver operating characteristic curves. Critical care.

[38] Lasko TA, Bhagwat JG, Zou KH, Ohno-Machado L (2005). The use of receiver operating characteristic curves in biomedical informatics. Journal of biomedical informatics.

[39] Vapnik, V. N. & Vapnik, V. *Statistical learning theory*, vol. 1 (Wiley New York, 1998).

[40] Hsu, C.-W., Chang, C.-C., Lin, C.-J. *et al*. A practical guide to support vector classification (2003).

[41] Keerthi SS, DeCoste D (2005). A modified finite newton method for fast solution of large scale linear svms. In Journal of Machine Learning Research.

[42] Combaz A (2013). A comparison of two spelling brain-computer interfaces based on visual p3 and ssvep in locked-in syndrome. PloS one.

[43] Combaz A (2012). Towards the detection of error-related potentials and its integration in the context of a p300 speller brain–computer interface. Neurocomputing.

[44] Di Russo F, Spinelli D (1999). Electrophysiological evidence for an early attentional mechanism in visual processing in humans. Vision research.

[45] Di Russo, F., Teder-Sälejärvi, W. A. & Hillyard, S. A. Steady-state vep and attentional visual processing. The cognitive electrophysiology of mind and brain (Zani A, Proverbio AM, eds) 259–274 (2002).

[46] Nakanishi M, Wang Y, Wang Y-T, Mitsukura Y, Jung T-P (2014). A high-speed brain speller using steady-state visual evoked potentials. International journal of neural systems.

[47] Wolpaw JR, Ramoser H, McFarland DJ, Pfurtscheller G (1998). Eeg-based communication: improved accuracy by response verification. IEEE transactions on Rehabilitation Engineering.

[48] Wolpaw JR, Birbaumer N, McFarland DJ, Pfurtscheller G, Vaughan TM (2002). Brain–computer interfaces for communication and control. Clinical neurophysiology.

[49] Volosyak, I., Valbuena, D., Luth, T. & Gräser, A. Towards an ssvep based bci with high itr. *IEEE Trans. Biomed. Eng*. (2010).

[50] Chen X, Chen Z, Gao S, Gao X (2014). A high-itr ssvep-based bci speller. Brain-Computer Interfaces.

[51] Lin, K., Chen, X., Huang, X., Ding, Q. & Gao, X. A hybrid bci speller based on the combination of emg envelopes and ssvep. In *Applied informatics*, vol. 2, 1 (Springer Berlin Heidelberg, 2015).

[52] Chen X (2015). High-speed spelling with a noninvasive brain–computer interface. Proceedings of the national academy of sciences.

[53] Kübler A, Neumann N, Wilhelm B, Hinterberger T, Birbaumer N (2004). Predictability of brain-computer communication. Journal of Psychophysiology.

[54] Kübler A, Birbaumer N (2008). Brain-computer interfaces and communication in paralysis: Extinction of goal directed thinking in completely paralysed patients?. Clinical neurophysiology.

[55] Brunner C, Allison B, Altstätter C, Neuper C (2011). A comparison of three brain-computer interfaces based on event-related desynchronization, steady state visual evoked potentials, or a hybrid approach using both signals. Journal of neural engineering.

[56] Pruzek, R. M. High dimensional covariance estimation: Avoiding the ‘curse of dimensionality’. In *Proceedings of the First US/Japan Conference on the Frontiers of Statistical Modeling: An Informational Approach*, 233–253 (Springer, 1994).

[57] Schoukens, J. & Pintelon, R. *Identification of linear systems: a practical guideline to accurate modeling* (Elsevier, 2014).

